# Characterization of silicon carbide diodes as cost‐effective active detectors for proton UHDR dosimetry

**DOI:** 10.1002/mp.17986

**Published:** 2025-07-15

**Authors:** Ivan Lopez Paz, Celeste Fleta, Paula Ibáñez, Ángela Henao, Daniel Sanchez‐Parcerisa, Adrián Zazpe, Ines del Monte‐Garcia, Consuelo Guardiola

**Affiliations:** ^1^ Institute of Microelectronics of Barcelona, IMB‐CNM (CSIC) Barcelona Spain; ^2^ Grupo de Física Nuclear & IPARCOS Universidad Complutense de Madrid Madrid Spain

**Keywords:** FLASH radiotherapy, ultra high dose rate, dosimetery, silicon carbide diodes

## Abstract

**Background:**

Proton therapy allows for better localization of the dose distribution in the tumor. In addition, the FLASH effect can be exploited to reduce the toxicity to healthy tissue by using short‐pulsed treatment at ultra‐high dose rates (UHDR). Such intense radiation conditions have limited options for active detectors for dosimetry.

**Purpose:**

Silicon carbide‐based diodes are proposed as a cost‐effective alternative to diamond detectors for dosimetry in UHDR.

**Methods:**

Two new SiC diodes designed and fabricated at the Institute of Microelectronics of Barcelona (IMB‐CNM) were exposed to 20 μs proton low‐energy pulses with up to 25 Gy per pulse to verify the capability of this technology to function under UHDR radiation at the Center for Microanalysis of Materials (CMAM) facility. The response of a single diode was correlated to the dose per pulse measured with calibrated EBT4 radiochromic films. Likewise, a 2 × 2 dosimeter matrix mounted on a motorized stage system was used as a proof‐of‐concept for a large array dose monitor under construction, by scanning the response of each of its pixels as a function of position with respect to the beam, compared against calibrated radiochromic films. Finally, the same device was exposed to varying pulse lengths, while connected to a current‐to‐voltage amplifier and an oscilloscope in order to measure the pulse structure.

**Results:**

First, the single diode showed a good dose rate linearity. No indication of saturation was observed even at the highest dose per pulse (DPP) of 25 Gy. This was observed even after over‐exposure of 52 kGy of 7 MeV protons, although its response lowered to 31% of the initially measured value. Second, the beam profiles observed by the pixelated detector were consistent with those of the reference measurements. Finally, the full width half maximums of the pulses observed by the pixels show good correlation with the pulse width of the beam.

**Conclusions:**

The SiC detectors developed at IMB‐CNM were able to withstand and accurately measure the dose under FLASH compatible beam characteristics, even in the case of low‐energy protons (7 MeV). The pixelated device showed promising results for a full array monitor for quality assurance, and the capability of time‐resolved pulse measurements, the latter after optimization of the electronics.

## 1 INTRODUCTION

Since 2014 there has been an increasing interest on the study of the biological FLASH effect^1^ in the radiotherapy (RT) research field, due to the differential response to radiation between tumorous and healthy tissue is increased by delivering the radiation treatment in short pulses (<100 ms) at very high dose rates (>40 Gy/s).^2^ These dose delivering conditions are referred to Ultra‐High Dose Rate (UHDR), as opposed to those dose rates in external beam of photons, where the treatments are performed continuously at a low rate (∼0.05 Gy/s). In particular, FLASH RT can reduce normal tissue damage while maintaining tumor response compared with conventional RT. Besides the FLASH biological effect, the UHDR beam modality allows to reduce the total treatment times and the organ movement uncertainties, which could benefit particularly the lung RT cases. The FLASH effect has been observed using UHDR beams of electron,^2^, ^3^, ^4^ photons^5^ and protons^6^, ^7^ in several tissue types, demonstrating a significant improvement in the TCP/NTCP ratio,^7^, ^8^, ^9^, ^10^ that is, increasing the therapeutic window. Preliminary results have also shown that FLASH RT could open up new treatment options for patients with radioresistant tumors. In addition, proton FLASH RT can mitigate delayed detrimental effects.^6^ Indeed, the first FLASH proton therapy clinical trial (FAST‐01) using single transmission fields has reported favorable results from the Cincinnati Children's Hospital Medical Center (CCHMC), USA,^11^ with an efficacy comparable with conventional RT and was followed by an ongoing trial (FAST‐02).^12^ However, there is still a considerable effort developing or adapting the corresponding accelerators to allow for the delivery of dose rates compatible with FLASH biology effects,^13^ for example, increasing the magnet scanning speed or the beam currents (80–600 nA) to deliver >40 Gy/s, reconsidering the energy‐switching for 3D ridge filters, i.a.^14^

Moving towards UHDR clinical translation requires robust standardized dosimetry protocols. However, enabling dosimetry tools for UHDR treatments is challenging given the high intensities and fast pulses.^15^, ^16^, ^17^ Currently, the leading methods for dose measurement primarily rely on passive dosimeters, such as radiochromic films,^18^ alanine,^19^ and TLDs.^20^ For example, EBT‐XD films are dose‐rate independent beyond 200 Gy/s,^21^ although the quenching effect must be taken into account under proton or higher LET fields. Overall, these passive dosimeters often require hours or even days to obtain accurate dose readings.

The traditional active detection techniques are not applicable to FLASH therapy due to their susceptibility to the extreme dose rates.^22^, ^23^ International guidelines recommend ion chambers for absorbed dose measurements,^24^ but at UHDR dose levels, ion recombination in the chamber cavity are significantly increased, leading to a sharp decline in ion collection efficiency^23^, ^24^, ^25^ and thus resulting in a dose rate dependence. A few studies have addressed these challenges,^26^, ^27^ with Gómez et al.^28^ recently developing the first ultra‐thin parallel plate ionization chamber with minimal ion recombination. Likewise, the European Joint Research Project UHDpulse aimed to develop metrological techniques and to establish a Code of Practice.^16^ Other detector technologies, such as calorimeters, are being studied by other groups.^29^, ^30^

In this line, solid state detectors based on silicon have been studied for active dosimetry,^31^, ^32^ although they show lower resistance to radiation with respect to other semiconductors, such as diamond. This carbon allotrope is becoming a standard for UHDR dosimetry.^33^, ^34^, ^35^, ^36^ For example, Marinelli et al.^33^ reported that several diamond Schottky diodes showed a linear charge response up to a dose of 26 Gy/pulse with dose rates of approximately 1 kGy/s when exposed to 7–9 MeV electron beam pulses (from 0.5 up to 4 μs). Nevertheless, diamond‐based diodes have the drawbacks of the high cost of the material and limited wafer availability.

A cost‐effective alternative is silicon carbide (SiC), a wide‐bandgap semiconductor with higher radiation resistance than silicon. SiC is currently available in 6 or 8‐in. high quality wafers, allowing for scale production. The lower radiation sensitivity of SiC (425 pC/($\text{mGy}\cdot {\mathrm{mm}}^{3}$)) with respect to silicon (644 pC/($\text{mGy}\cdot {\mathrm{mm}}^{3}$)) reduces the possibility of signal saturation in high intensity beams. Regarding these features, 4H‐SiC diodes based on implanted PN‐junctions have been designed, manufactured, and characterized by the Institute of Microelectronics of Barcelona (IMB‐CNM, CSIC) over the last years for high‐energy physics applications. Initially, 4‐quadrant SiC diodes for beam monitoring were developed for synchrotron applications on 30 μm epitaxial wafers, showing the improved radiation hardness of the devices^37^, ^38^ with respect to their silicon counterparts by demonstrating charge collection after a 2.5 × 10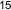 ${\mathrm{cm}}^{-2}$ proton irradiation. This study was followed by tests for plasma diagnostics using 3.5 MeV alphas (at 10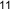 ${\mathrm{cm}}^{-2}s^{-1}$ fluence) while operated up to 450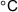, showing a better than 2% energy resolution under these conditions.^39^, ^40^ Later, IMB‐CNM fabricated SiC diodes with a reduced metallic contact area that interfaces with the active area using a graphene layer^41^ to reduce multiple scattering effects. These samples were characterized in conventional RT with 6 MV photons and a dose rate up to 6 Gy/min, yielding a linearity better than 2%.^42^, ^43^ Finally, the same devices were characterized with 20 keV synchrotron x‐rays.^44^ Based on this technological background, novel 3 μm epitaxial SiC diodes have been designed and fabricated at IMB‐CNM for dosimetry in UHDR beams. They were tested at null bias under 20 MeV electron beams with doses up to 11 Gy per pulse (pulse durations from 3 to 0.5 μs), that is, 4 MGy/s, maintaining a linearity better than 3%,^45^ the highest reported with silicon carbide so far. The PDD SiC performances at this pulse range were comparable to reference PTW flashDiamond results. The sensitivity reduction after 100 kGy accumulated dose was 2%. Likewise, this 3 μm epitaxial SiC diode, operated without bias, was able to follow adequately the temporal structure of those electron pulses in the UHDR regime. These results have been later replicated by other groups^46^ demonstrating the exceptional performance of silicon carbide for relative dosimetry in UHDR beams.

In this work, both a single and a 2 × 2 array of 3 μm epitaxial SiC diodes from IMB‐CNM are studied under 7 MeV proton UHDR beams with 20 μs pulses and a 1.54 ms pulse distance at the Center for Microanalysis of Materials (CMAM).^47^ The linearity of a single SiC diode with the dose per pulse is studied. Likewise, the performance of a SiC 2×2‐array proof‐of‐concept for position resolution and time structure measurements is explored for the first time.

## 2 MATERIALS AND METHODS

### 2.1 SiC detectors

**FIGURE 1:**
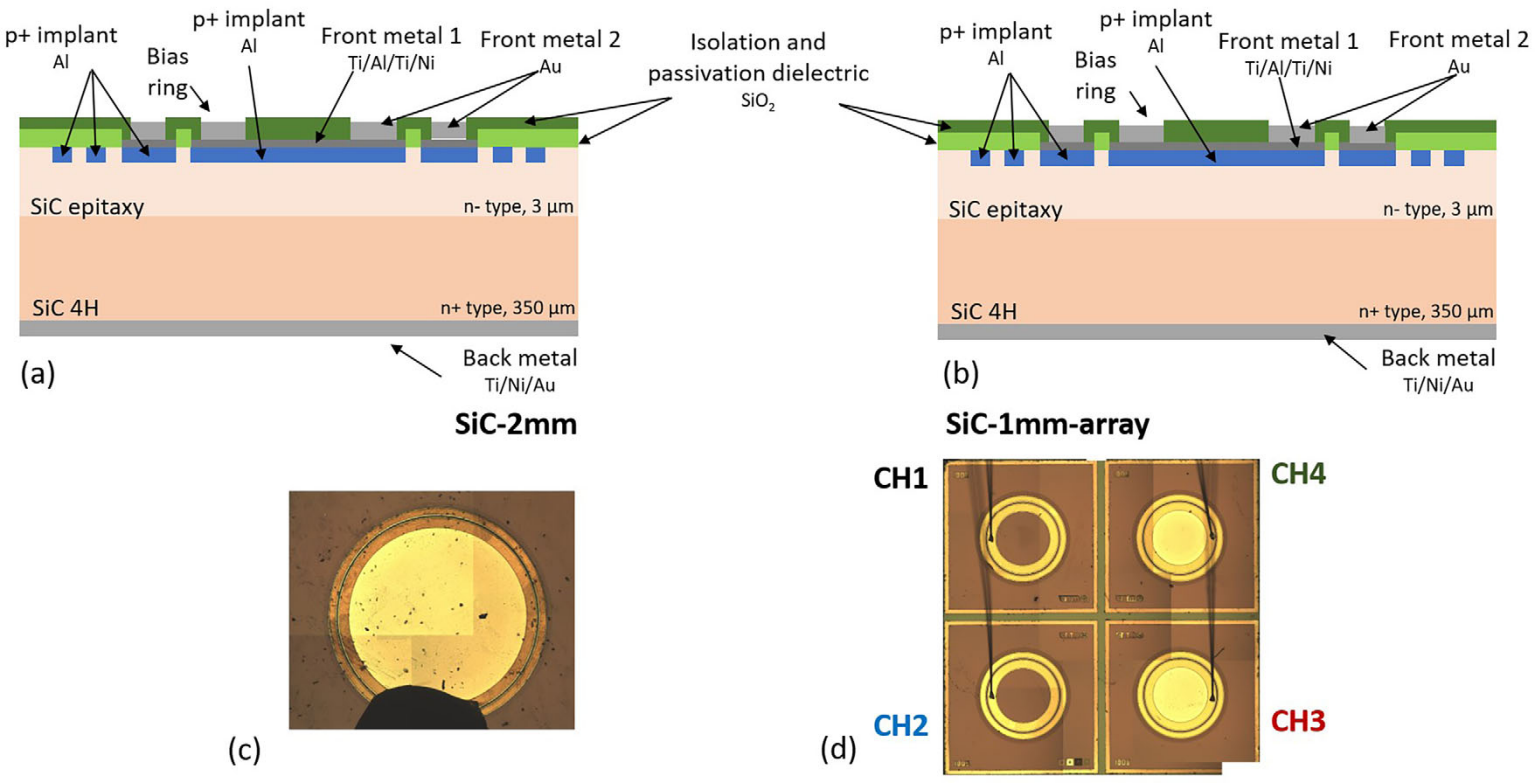
Cross‐sections. (a) Sketch of cross‐sections of the main components of the SiC diodes with fully metalized active area and (b) metalized only in the periphery. (c) Pictures of the SiC‐2 mm and (d) SiC‐1 mm‐array. In the latter, the wire bonds used for interconnection to the electronics are visible (grey vertical lines).

Silicon carbide diodes were designed and fabricated at the IMB‐CNM on 3 μm high resistivity epitaxial 4H‐SiC wafers on a 350 μm low resistivity substrate. The PN junction is achieved by aluminum implantations followed by a high temperature annealing. To contact the junction area, a combination of Ti/Al/Ti/Ni metal layers was deposited, followed by a Ti/Au layer in the periphery region of the diodes to allow for wire bonding. The wafer was otherwise covered by a dielectric layer of ${\text{SiO}}_{2}$ for protection and electrical isolation. The back side was fully metalized with a Ti/Ni/Au metal stack. Further details on the fabrication process are reported elsewhere.^45^ Figures 1a,b shows a cross section of the structures.

Among the structures produced in the wafer, circular diodes of 2.2 mm diameter (SiC‐2 mm) and a 2 × 2 arrays of 1 mm diameter diodes (SiC‐1 mm‐array) were fabricated (see Figure 1c,d).

In the SiC‐1 mm‐array sample, the pixels were separated by a 2.2 mm pitch in either direction. While the SiC‐2 mm active area was fully covered by the first metal stack (see Figure 1a), the pixels in the matrix had either the full active area metalized or only the periphery region (see Figure 1a,b). However, the difference in metalization strategy did not show a significant impact on the response with protons during the experiment, as expected due to the low energy loss of protons in that energy range in the metallic layers. The devices under test (DUT) were mounted on printed circuit boards with either 1 or 4 coaxial connections, and wirebonded for connectivity (Figure 1c,d). Thus, the SiC‐2m (single channel DUT) was used to verify the linearity of the diode response, while the SiC‐1 mm‐array (four channel DUT) was selected as a demonstrator for a possible beam monitor.

### 2.2 Experimental setup

The DUTs were characterized with 7 MeV proton UHDR beams at the Center for Microanalysis of Materials (CMAM) external beam line. A Faraday Cup located before the beamline exit monitors the downstream beam current. This was used for monitoring possible intensity fluctuations for dose determination. The beam is pulsed in microseconds mode using a chopper that deflects the particle beam in three stages, to ensure that the resulting beam exits centered without deviation and working in burst mode. The deflection of the beam is realized by applying an electric field, and the beam is able to reach the beam end‐point otherwise. This system is controlled with a function generator, which allows the temporal structure of the pulse train (i.e., pulse width, frequency and number of pulses in the train) to be controlled. For this experiment, the voltage applied to the deflector was 500 V, keeping the beam deflected at all times until the beam output was triggered by the function generator. The pulse length used was 20 μs with an inter‐pulse time of 1.54 ms, matching one of the proton pulse structures that has been shown in the literature to trigger the FLASH effect.^48^ The settings utilized to generate such a pulsed structure on the function generator are displayed in Table 1, which led to the pulsed beam characteristics summarized in Table 2.

**TABLE 1:** Settings to generate the pulsed beam.

Frequency (Hz)	Amplitude (V)	Offset (V)	Duty cycle (%)	Edge time (ns)
641	5	5	1.3	8

**TABLE 2:** Nominal pulsed beam characteristics.

Pulse width	Beam current	Dose per pulse	Instantaneous	Mean dose rate
(μs)	(nA)	(Gy)	dose rate (MGy/s)	(kGy/s)
20	232–9310	0.4–25.6	0.2–1.3	2.7–166.2

*Note*: Beam currents were measured by the beam line Faraday Cup, and the doses were obtained from the radiochromic films (see Section 2.3).

**FIGURE 2:**
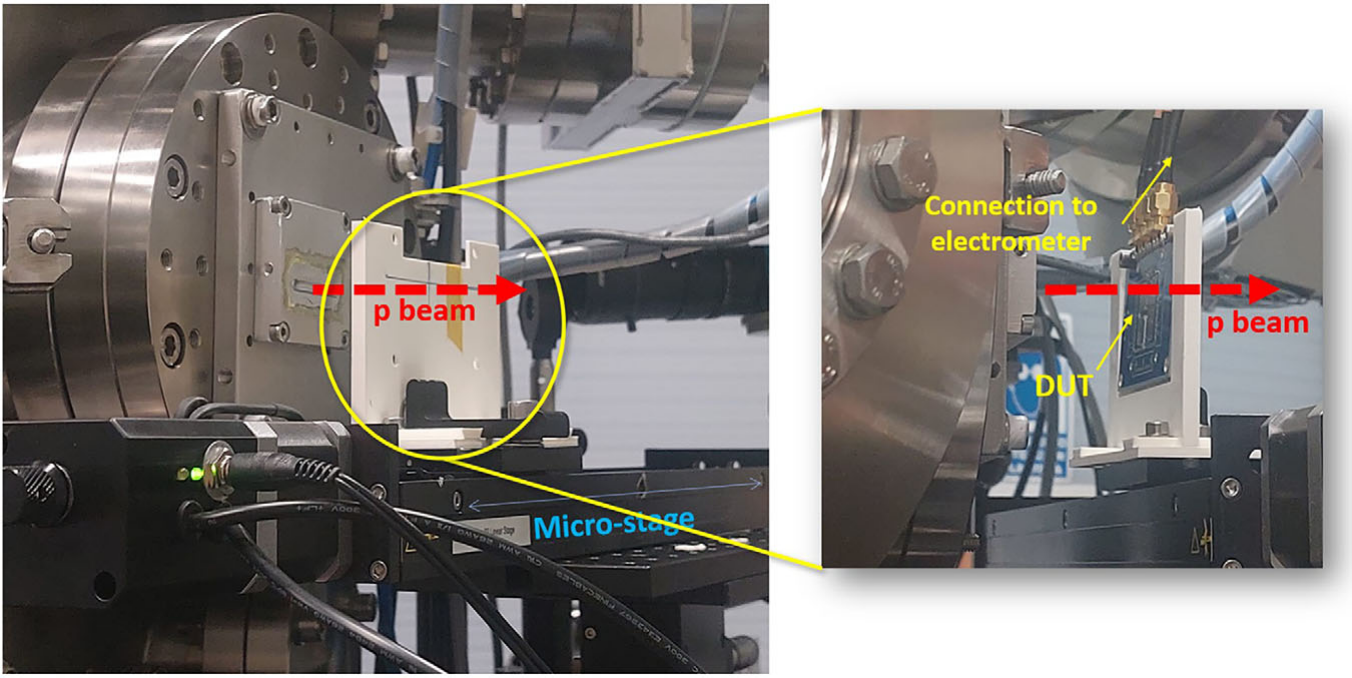
Picture of the experimental setup.

Both DUTs were mounted on 3D‐printed frames, which were attached to a micrometer‐precision single‐axis stage (see Figure 2), with the detectors positioned at 40 ± 1 mm from the beam exit window. This allowed a precise horizontal positioning, which was adjusted in 100 μm steps. To ensure that the diode active area was fully covered by the beam aperture, the height of the stage was adjusted manually by aligning the diodes with radiochromic films attached on them (see Section 2.3).

Three irradiation studies were performed to quantify the (a) dose rate linearity, and the (b) position and (c) time resolution of the SiC devices.

(a) Dose rate linearity with the SiC‐2 mm detector: the diode was connected to a PTW Unidos E electrometer. In order to read out the accumulated charge response after exposure to 20 μs beam pulses, 3 × 100 nF capacitors in parallel (i.e., 300 nF) were connected between the diode and the electrometer. Data taking started before exposure, and the accumulated charge was recorded every second. To remove background from the data, the charge accumulated before the pulse train arrival was removed from the integrated charge. The diode response was correlated to the dose measured with EBT4 radiochromic films (see section 2.3).
(b) Position resolved with the SiC‐1 mm‐array: the array was mounted on a micrometer precision stage to, first, find the beam spot, and second, scan the irradiation zone with micrometer steps. The four pixels of the device were connected to a 4‐channel electrometer, with 1 kSample/s readout capability, and a 100 nF capacitor in parallel. Three approaches were used to measure the beam profile. First, an EBT4 radiochromic film was exposed before and after the measurement set. Second, the 4‐channel array was continuously moved at 200 μm/s in the horizontal direction, while being exposed to 1 Hz pulses, resulting in current peaks every 200 μm for each of the 3–4 sweeps (“continuous”) as measured from the 4‐channel electrometer. Notice that the current in the detector is proportional to the instantaneous dose rate and therefore, for constant pulse widths, also proportional to dose per pulse. Finally, over the same horizontal range and in steps of 500 μm, the integrated charge was measured from 10 proton pulses separated by 1.54 ms, three times for each position to collect statistics (“discrete”). When measuring the beam profile, the “discrete” measurement is slow since it requires single deliveries after every sensor displacement with the stage, but it is more robust since the charge is collected with enough time to ensure full integration and readout, while the “continuous” measurement is faster as the sensor is continuously moved at a speed of 200 μm/s, but these readings are more sensitive to charge variations and uncertainties associated to the readout time. Nevertheless, using both strategies allows to demonstrate the consistency of the detector readings.
(c) Time resolved with the SiC‐1 mm‐array: an in‐house multi‐channel current amplifier was designed and produced to allow for time structure measurements of the pulse. Two out of four pixels in the SiC‐1 mm‐array detector were connected to the amplifier, which was set to a 10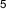 V/A amplification. The output was connected to a 5 GSa/s Yokogawa DLM6000 oscilloscope. By varying the pulse length in the range of 5–30 μs, the variation of the pulse time structure could be observed.

### 2.3 Reference dosimetry

Calibrated EBT4 GAFChromic radiochromic films^49^ were placed directly in front of the detector for each measurement set. The number of pulses was chosen so that the accumulated dose remained below 12–15 Gy to avoid film saturation. Therefore, films were irradiated with one pulse at larger doses, while for the lower doses, 10 pulses were used. The downstream accelerator beam current was recorded before each irradiation. Due to the dose limitation of the films, when the accumulated dose exceeds it, the accelerator current is used to extrapolate the dose per pulse.

The response of the radiochromic films is affected by the proton energy, which is accounted for by a relative efficiency (RE)^50^ factor. In order to determine the RE factor, the energy of the protons reaching the active area of the films is obtained by simulation with the GATE Monte Carlo code^51^, ^52^ to be 6.52 ± 0.03 MeV. Therefore, the RE for this set‐up is RE = 0.86.

The dose was estimated by averaging the optical density in a region of interest (ROI) with the same area as the detector and normalizing by the number of pulses. The ROI was centered in the maximum dose region, since the detector was aligned in 100 μm steps along the horizontal direction by maximizing its response. For each data point taken by the DUT, the dose measured with the film was corrected for the difference in beam current monitored by the accelerator. Thus, the final dose for each point $D_{m}$ is given by the expression:

(1) $$D_{m}=\frac{1}{RE}\frac{I_{m}}{I_{film}}D_{film},$$

where $D_{film}$ is the accumulated dose measured with the radiochromic film, and $I_{film}$ and $I_{m}$ are the beam currents measured before exposure of the film and the DUT, respectively.

**FIGURE 3:**
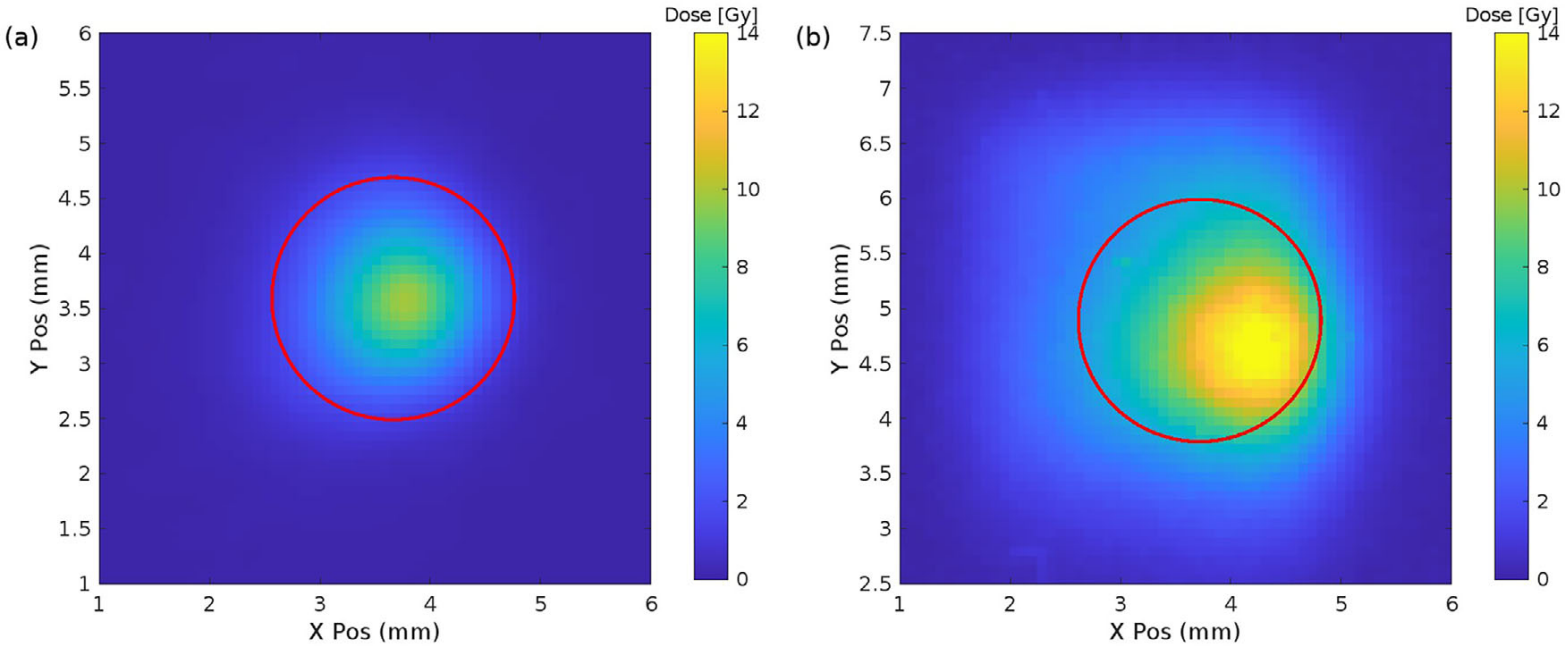
Beamspots. Dose distribution obtained from radiochromic films and with the circular 2 mm diameter ROI (red circle) for the initial measurements (a) and after higher irradiation exposure (b).

Notice that the inhomogeneity of the beam (see Figure 3) is the main uncertainty in the dose, and it comes from the alignment accuracy. In order to account for possible beam position instabilities, a Monte Carlo simulation was performed where *N* = 1000 points were sampled with a σ = 200 μm, to estimate the dosimetric uncertainty related to film/diode relative positioning.

## 3 RESULTS

By adjusting the downstream beam current up to 14.5 μA, the dose rate at the beam exit was modified, yielding up to 25 Gy per pulse from the dosimetry. For each condition, the doses were measured as described in Sec. 2.3.

For the first measurement set (a), regarding the dose rate linearity of the SiC‐2 mm diode, the beam spot was kept Gaussian‐shaped with a 2 mm width (see Figure 3a). However, such a small beam size, comparable to the active area of the device under test, makes the dose determination challenging by increasing the uncertainties by alignment. The beam settings were adjusted by increasing the beam astigmatism (i.e., defocusing the beam in a quadrupole in the ion beam optics) in an attempt to broaden the beam profile. While this leads to a wider beam, the dose distribution becomes more inhomogeneous (Figure 3b).

**FIGURE 4:**
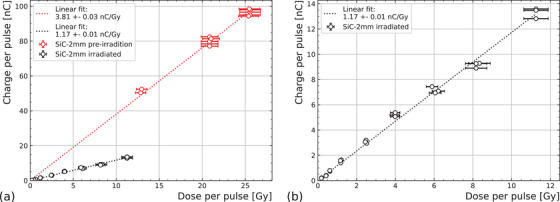
SiC cross section and duts. (a) Charge response as a function of dose per pulse measured with radiochromic films before and after exposure to 52 kGy. (b) Magnification of the charge response as a function of dose per pulse after irradiation.

Figure 4 shows the charge per pulse collected by the SiC‐2 mm diode as a function of the dose per pulse. Initially, the SiC‐2 mm sample was measured up to a 25 Gy per pulse, the DPP highest reported yet with silicon‐carbide diodes and with UHDR proton beams. In order to reach dosimetric readings above the radiochromic limits, the dose was extrapolated from 12.8 Gy using the Tandem beam current according to Equation (1). Further dose per pulse measurements would require different reference dosimeters due to film saturation, such as alanine. A linear regression model was applied between the dose and charge per pulse, showing a lineal behavior (*R* 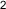 = 0.985) and yielding a response of (3.81 ± 0.03) nC/Gy, compatible with the theoretical sensitivity of 425 pC/($\text{mGy}\cdot {\mathrm{mm}}^{3}$) and consistent with smaller samples irradiated with electrons.^45^

It is worth noting that after the first measurement set, during re‐alignment, the detector was exposed to 52 ± 3 kGy, and the detector charge as a function of dose was re‐measured, as shown in Figure 4b. Post‐irradiation, the device kept linearity (*R* 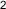
= 0.995) although with a lower sensitivity of (1.17 ± 0.01) nC/Gy, which corresponds to 31% of the original.

For the second measurement (b), the SiC‐1 mm‐array device was mounted at three different heights (*y* = 0, −1, and 1 mm, or positions 1, 2, and 3 respectively) with respect to the beam center position. Radiochromic films were irradiated before and after this set of measurements to cross‐check the beam profile with the measured by the detector.

**FIGURE 5:**
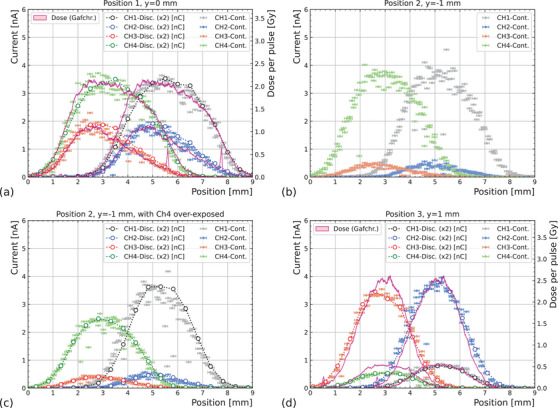
SiC position resolved. Position resolved measurements taken at 3 different detector heights (*y* = 0, −1, and 1 mm). When available, the dose profile measured with a radiochromic film is included (an artifact in the film produced a spike in the dosimetry for position 1 (a)). Two pixels (CH3 and CH4) were over‐exposed, and the position‐resolved scans were repeated.

Notice that the beam profile changed slightly over time possibly due to the warming up of the beam optics, and therefore the film measurements taken before and after the full set were not valid for comparison in position 2 (*y* = − 1 mm), as enough time had elapsed between measurements for the beam profile to change (due to time limitations, it was not possible to irradiate a reference film for position 2). Figure 5 shows the response of the device in the “continuous” and “discrete” scans, as well as the profile obtained by the radiochromic films before (position 1) and after (position 3) the scans. The beam profiles are consistent across measurements, showing the capability for beam monitoring at a position resolution better than 1 mm, thus proving the validity of the proof‐of‐concept. Only the pixels in CH3 and CH4 featured a fully metalized active area as opposed to CH1 and CH2 (see Figure 2d, bottom, right). However no significant difference in their response was observed between pixels positioned at the same height (CH1 vs. CH3 and CH2 vs. CH4), as seen in Figure 5a.

After the “continuous” scan at position 2 (*y* = −1 mm), pixels in CH4 and at lower extent CH3, were over‐exposed with a continuously pulsing proton beam during beam re‐alignment. The measurements were repeated to assess the relative loss in response, leading to approximately 69% and 88% of their original response, respectively. As consequence, in the final measurement set (position 3), the SiC measurements in CH4 and CH3 show a lower response than expected from the radiochromic films, while CH1 and CH2 fit the reference measurements.

**FIGURE 6:**
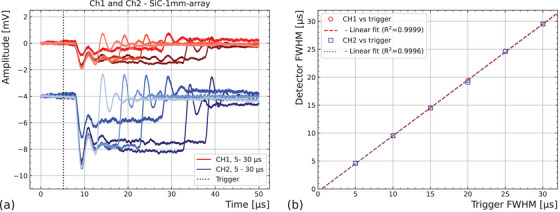
SiC time resolved. (a) Waveforms obtained after 10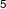 V/A amplification of pixel response at different beam pulse widths adjusted with a pulse generator. The different shades of red and blue reflect different pulse widths for CH1 and CH2, ranging from 5 to 30 μs in 5 μs steps (from lighter to darker). (b) Correlation between the FWHM of the pixel signals and the trigger pulse width.

Finally, the third measurement (c), CH1 and CH2 of the SiC‐1 mm‐array device were connected to the amplifier set with a gain of 10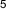 V/A, the output of which was connected to an oscilloscope. The beam was configured to supply about 2 Gy per 20 μs pulse before variation of the pulse width similar to that of the previous measurement (b), although the pixels were not aligned to the beam center. Figure 6a shows the response of each pixel to beam pulses from 5 to 30 μs. Amplitude differences between waveforms are attributed to instabilities in the beam intensity across measurements, since at the time, no device was available to obtain an independent measurement of the beam structures. Short oscillations are observed at either end of the pulses, which may be caused by impedance mismatch. The full width half maximum (FWHM) of each signal is correlated with the FWHM of the trigger pulse generator and shown in Figure 6 (*R* 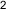 = 0.9999 and *R* 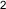 = 0.9996 for CH1 and CH2 respectively). With the amplification factor available, the signals reaching the oscilloscope were small, ranging from 1 to 4 mV after several meters of coaxial cable, which limits the signal‐to‐noise ratio. A higher amplification factor would improve the signal shaping by improving the signal‐to‐noise ratio.

## 4 DISCUSSION

Enabling dosimeters that deal with the dose rate dependence and the spatial‐ and time‐resolution under UHDR conditions is a technological challenge in the field. Although some passive detectors (TLD, OSLD, radiochromic films, and alanine) have shown dose rate independence, they require long‐time analysis and give only isolated measurements. On the other hand, diamond diodes have been successfully utilized for dosimetry in these conditions.^33^, ^34^, ^36^ However, detector‐grade diamond—characterized by minimal defect density to ensure long charge carrier lifetimes and high charge collection efficiency—is expensive and it is not available in large wafer sizes (larger than a few mm), and it is therefore difficult to scale for mass‐production or for large‐area dose monitors. In contrast, large high quality SiC wafers are now commercially available, making fabrication of detectors feasible, scalable, and cost‐effective.

The 2.2 mm SiC diode (SiC‐2 mm) presented in this work showed a good linear response versus the dose rate with 7 MeV protons and no indications of saturation even at 25 Gy per pulse. Similarly, after 52 kGy irradiation, it maintained the linear behavior up to the measured 11 Gy per pulse.

The response of the post‐irradiated device is estimated to be 31% of the original, owing to the creation of radiation‐induced defects in the crystal that act as traps or recombination centers.^38^ The averaged sensitivity loss with 7 MeV protons of the SiC‐2 mm is therefore ∼1.3%/kGy. The sensitivity loss of SiC diodes from the same production as the studied here has been examined under 2 MeV proton irradiation in a different experiment,^53^ yielding 1.34%/kGy up to the first 190 kGy accumulated dose, compatible with this work. For comparison, a commercial silicon diode dosimeter from PTW tested by Raffaele et al.^54^ shows a sensitivity loss of 4%–7%/kGy with 62 MeV protons. Other Si diodes showed even larger decreases in sensitivity (2%–10%/kGy) after proton irradiations.^55^, ^56^ Moreover, the dose accumulated by electrons has a much lower impact on the dosimetric sensitivity of semiconductors than that by protons. Indeed, the sensitivity loss with electrons (20 MeV) of this production of SiC diodes was measured to be 0.02%/kGy^45^ while commercial silicon dosimeters report 0.1%/kGy with 6 MV electrons.^57^

The good linearity with dose rate of the device, even after high radiation doses, indicates that its lifetime can be extended, provided periodic recalibrations are done to correct for the difference in sensitivity. In addition, as Jimenez‐Ramos et al.^53^ have demonstrated, after MGy levels of irradiation, the sensitivity no longer degrades while the diode remains fully functional. Therefore, no further dosimetric calibrations would be required beyond that point. Nevertheless, more experiments are foreseen, focused on the radiation damage and the energy dependence of the sensitivity loss, in order to provide a prescription for the use of the SiC technology for proton FLASH.

Besides, the dosimetry and time‐resolved measurement show that the implantation beam line at CMAM can produce FLASH‐like beam conditions:^48^ 20 μs proton pulses with dose per pulse exceeding 10 Gy. Although the beam spot size was small, making the dosimetry and large area exposure challenging, beam upgrades are possible to expand the proton source by for example, the addition of diffusion layers and an optimized adjustment of the beam optics. This facility would allow for further research of potential dosimeters.

With a 4 pixel matrix of 1 mm diameter SiC pixels, we could measure the beam profile with a good resolution, as all measurement strategies yield compatible results (Figure 5). This system serves as a proof‐of‐concept to show the viability of a dose monitor under development that consists in an array of 400 pixels with 600 μm diameter and a pitch of 1 mm mounted in a movable stage to scan the complete irradiation field, spanning up to 40 cm, if necessary. In most cases, the pixels on the extreme sides will be less exposed to radiation. Therefore, the same configuration would allow to assess the need for further calibration by comparing the response between inner and outer pixels, similar to Figure 5c.

The time structure of the beam was measured by means of a current‐to‐voltage transformer produced in‐house. The full‐width half‐maxima maxima measured from the pixels are consistent to that to the pulse generator used to structure the beam pulses (Figure 6b). An optimized electronics would allow to determine the instantaneous dose within each beam pulse for further beam monitoring.

## 5 CONCLUSIONS

New silicon carbide diodes of 2.2 mm diameter (SiC‐2 mm) and arrays of 1 mm diameter (SiC‐1 mm‐array) with 3 μm epitaxy have been designed and fabricated at IMB‐CNM for a cost‐effective solution for FLASH on‐line dosimetry. The single diode technology has been tested and validated with UHDR electrons.^45^ In this paper, the performances of both configurations under low‐energy proton beams (7 MeV) are reported for the first time.

The SiC‐2 mm diode was able to maintain a linear behavior up to 25 Gy per pulse. After 52 kGy exposure, the detector lost responsivity ‐ which is hardly reported in FLASH dosimetry studies ‐ while maintaining a lineal dose rate response.

Therefore, after such high radiation doses, its lifetime can be easily extended by periodic recalibrations, as it maintains the same functionality under FLASH conditions. Moreover, thanks to the fast SiC response, if it is joined to a suitable electronics, it is possible to determine the intra‐pulse characteristics.

The capacity of using SiC arrays to have dose maps was assessed with a first SiC‐1 mm‐array proof‐of‐concept. It was placed on a motorized micro‐stage, obtaining current measurements with better than 1 mm resolution. The pixel diameter is not limited by the technology. Indeed, the fabrication of smaller pixel (30 μm diameter) dosimeters has been realized within the Institute.^53^

Results demonstrated the viability of the SiC technology developed at CNM for proton FLASH dosimetry and the possibility of arranging these SiC diodes in a 2D‐matrix for obtaining dose maps. This would enable the assessment of dosimetry distributions under FLASH RT.

## CONFLICT OF INTEREST STATEMENT

The authors declare no conflicts of interest.
